# Anti-Oxidative and Anti-Proliferative Activity on Human Prostate Cancer Cells Lines of the Phenolic Compounds from *Corylopsis coreana* Uyeki

**DOI:** 10.3390/molecules18054876

**Published:** 2013-04-24

**Authors:** Manh Heun Kim, Sung Yi Ha, Myeong Hwan Oh, Han Hyuk Kim, So Ra Kim, Min Won Lee

**Affiliations:** College of Pharmacy, Chung-Ang University, Seoul 156-756, Korea

**Keywords:** *Corylopsis coreana*. Uyeki, 1,1-diphenyl-2-picrylhydazyl radical, superoxide scavenging, LNCaP, DU145

## Abstract

Fifteen phenolic compounds, including three caffeoyl derivatives, four gallotannins, three ellagitannins and five flavonoids, were isolated from an 80% MeOH extract of the leaves of *Corylopsis coreana* Uyeki (Korean winter hazel; CL). The anti-oxidative activities [1,1-diphenyl-2-picrylhydrazyl (DPPH) radical scavenging activity and xanthine oxidase superoxide scavenging activities (NBT)] and the anti-proliferative activity on human prostate cancer cell lines (DU145 and LNCaP) were also evaluated.

## 1. Introduction

*Corylopsis coreana* Uyeki (Korean winter hazel; CL) belongs to the Hamamelidaceae or witch hazel family, [1]. CL is cultivated as an ornamental plant in South Korea. Some species of the genus *Corylopsis*, such as *Hamamelis virginiana* (witch hazel) have been a folk medicine for the treatment of irritated skin and inflammatory disease [2] and witch hazel bark are widely used in skin care products for sun burn, atopic eczema, *etc*. [3]. It has been reported that hamamelitannin and simple phenolics were isolated from the bark of witch hazel [4], and their anti-mutagenic activities and anti-cancer activities against various forms of cancer were also reported [5]. This paper describes the isolation of compounds from the leaves of CL and evaluation of its anti-oxidative activities and the anti-proliferation properties of the isolated compounds.

## 2. Results and Discussion

### 2.1. Isolation and Identification

**Scheme 1:**
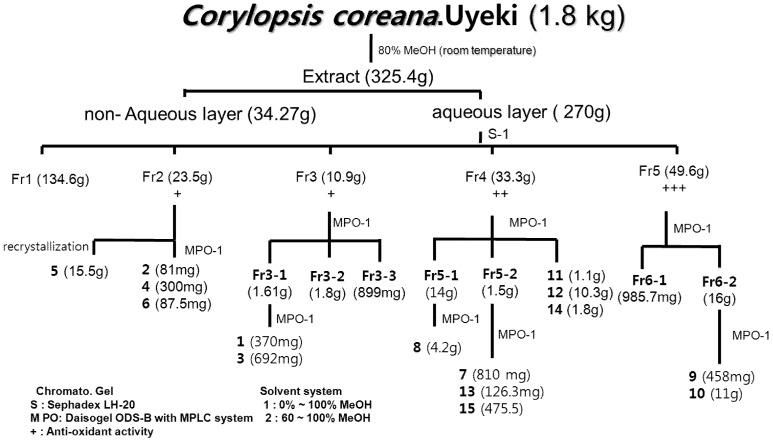
Extraction and Isolation of Compounds **1**–**15** from CL.

**Figure 1:**
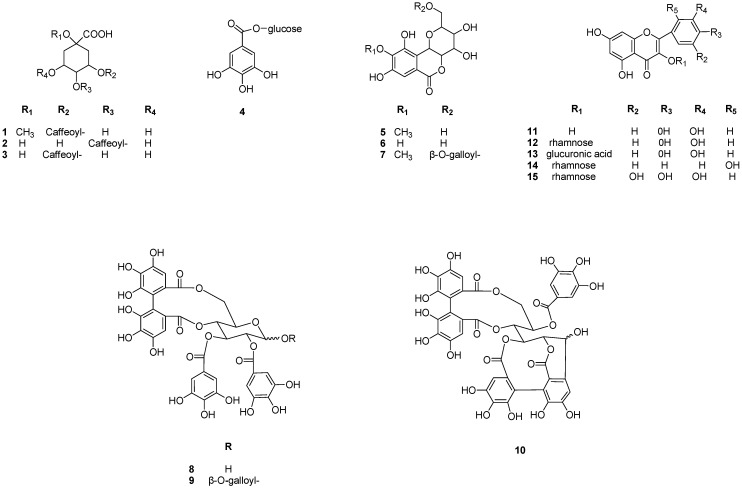
Structures of compounds **1**–**15** isolated from CL.

Anti-oxidant activity-guided chromatographic fractionation of leaves of CL afforded fifteen phenolic compounds (Scheme 1 and Table 1). The structures of compounds **1**–**15** were identified as 3-caffeoylquinic acid methyl ester (**1**) [6], 4-caffeoylquinic acid (**2**) [6], 3-caffeoylquinic acid (**3**) [6], 3-*O*-galloyl-*β*-d-glucopyranoside (**4**) [7], bergenin (**5**) [8], nor-bergenin (**6**) [8], 11-galloylbergenin (**7**) [9], tellimagrandin I (**8**) [10], tellimagrandin II (**9**) [11], casuarinin (**10**) [12], quercetin (**11**) [13], quercitrin (**12**) [14], quercetin 3-*O*-*β*-d-glucuronide (**13**) [15], datiscetin 3-*O*-*β*-d-rhamnpyranoside (**14**) [16] and myricetin 3-*O*-*β*-d-rhamnopyranoside (**15**) [17], respectively (Figure 1), by comparing the spectral (MS, NMR) data with the values reported in the literature.

**Table 1:** DPPH radical scavenging and superoxide scavenging activities of each fraction from CL.

Fractions	DPPH radical scavenging activity SC_50_ (mg/mL)	Superoxide scavenging activity SC_50_ (mg/mL)
**1**	>100 ^e^	59.02 ± 1.07 ^d^
**2**	43.16 ± 0.48 ^d^	23.64 ± 0.92 ^c^
**3**	20.26 ± 0.43 ^c^	13.35 ± 1.09 ^b^
**4**	11.22 ±0 24 ^b^	5.89 ± 0.91 ^a^
**5**	5.89 ± 0.03 ^a^	2.42 ± 0.73 ^a^
**Ascorbic acid**	6.68 ± 0.11 ^a^	-
**Allopurinol**	-	1.70 ± 0.92 ^a^

Values represent means ± S.D*.* of three determinations. Values bearing different superscripts in the same column are significantly different (*p* < 0.05).

### 2.2. Anti-Oxidative Activity (DPPH Radical, Superoxide Scavenging Activity)

Oxidative damage appears to be related to various diseases and inflammation conditions [18,19]. Polyphenols reduce oxidative stress and provide activity associated with anti-cancer, anti-allergic, and anti-inflammatory effects [20]. In order to evaluate the anti-oxidative activities of the compounds **1**–**10** from CL, their DPPH radical [21] and superoxide scavenging activities were tested [22]. Among the compounds **1**–**10** from CL, **8**, **9** and **10** showed more potent free radical scavenging activity than ascorbic acid (SC_50_ = 8.22 ± 0.64 μM), with SC_50_ values of 3.12 ± 0.05, 2.97 ± 0.04 and 3.29 ± 0.26 μM (*p* < 0.05) (Table 1), due to presence of a galloyl groups plus an HHDP group in their structures, which plays an important role in anti-oxidation by donating hydrogen atoms to active free radicals [23]. In addition, **8**, **9** and **10** also showed more potent superoxide scavenging activity than allopurinol (SC_50_ = 2.39 ± 0.09 μM), with SC_50_ values of 0.29 ± 0.69, 0.09 ± 0.02 and 0.16 ± 0.02 μM (*p* < 0.05) (Table 2).

### 2.3. Cell Viability and Inhibition of Cancer Cell Proliferation

**Figure 2:**
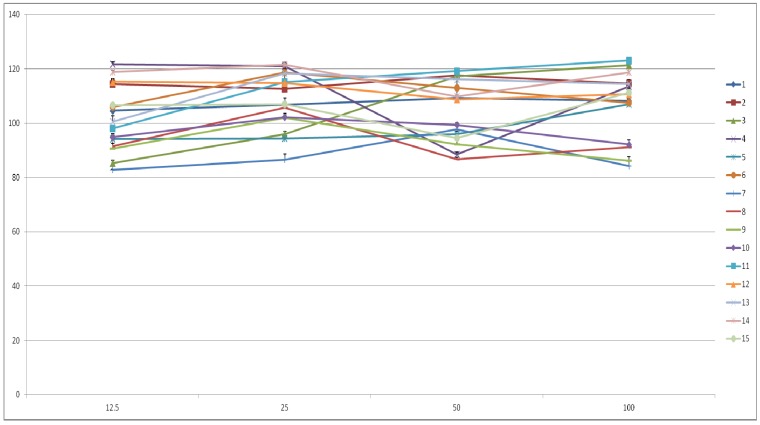
Cell viability of compounds **1**–**15** from CL on RAW 264.7 cell lines.

The cell viability was measured using the MTT assay (Figure 2), which is based on the mitochondria-dependent reduction of MTT to formazan [24]. In order to evaluate the anti-proliferation activities of the compounds **1**–**15** from CL, cell viability were tested on DU145 and LNCaP prostate cancer cells. The anti-proliferative effects of hydrolysable tannins in sarcoma cells and HeLa cells were reported [25] and the functional groups the hydrolysable tannins are also important factors determining their anti-proliferation activity [26]. Among the ellagitannins **8**–**10**, compound **10** showed higher androgen sensitive anti-proliferation activity, suggesting the importance of the HHDP group. Since **9** was more potent than **10**, the presence of both HHDP and galloyl groups might be necessary. Compound **9** was also more potent than **8**, suggesting the importance of a galloyl group in the C-l position [27]. The compounds **8**, **9** and **10** inhibited the proliferation of both DU145 and LNCaP prostate cancer cells (Table 3).

**Table 2:** DPPH radical scavenging and superoxide scavenging activities of each compounds **1**–**15** from CL.

Compounds	DPPH radical scavenging activity SC_50_ (μM/mL)	Superoxide scavenging activity SC_50_ (μM/mL)
**1**	23.05 ± 0.92 ^d,e^	13.47 ± 0.05 ^b^
**2**	51.49 ± 0.77 ^g^	11.71 ± 0.36 ^b^
**3**	21.65 ± 0.72 ^d,e^	22.68 ± 0.35 ^d^
**4**	13.41 ± 0.10 ^c^	10.81 ± 0.07 ^c^
**5**	>100 ^i^	>100 ^e^
**6**	36.34 ± 0.93 ^f^	13.68 ± 0.74 ^b, c^
**7**	21.63 ± 0.70 ^e^	3.85 ± 0.03 ^a^
**8**	3.12 ± 0.05 ^a^	0.29 ± 0.69 ^a^
**9**	2.97 ± 0.04 ^a^	0.09 ± 0.02 ^a^
**10**	3.29 ± 0.26 ^a^	0.16 ± 0.02 ^a^
**11**	49.09 ± 0.92 ^g^	12.18 ± 0.14 ^b,c^
**12**	60.96 ± 0.70 ^h^	12.43 ± 0.24 ^b,c^
**13**	17.06 ± 0.15 ^d^	3.17 ± 0.78 ^a^
**14**	36.87 ± 0.44 ^f^	2.59 ± 0.75 ^a^
**15**	19.45 ± 0.05 ^d,e^	1.45 ± 0.24 ^a^
**Ascorbic acid**	8.22 ± 0.64 ^b^	-
**Allopurinol**	-	2.39±0.09 ^a^

Values represent means ± S.D. of three determinations. Values bearing different superscripts in the same column are significantly different (*p* < 0.05).

**Table 3:** Anti-proliferative effects of compounds **1**–**15** from CL on LNCaP and DU145 cancer cell lines.

Compounds	Anti-proliferative activity of LNCaP cancer cell line IC_50_ (μM/mL)	Anti-proliferative activity of Du-145 cancer cell line IC_50_ (μM/mL)
**1**	>100 ^c^	>100 ^a^
**2**	>100 ^c^	>100 ^a^
**3**	>100 ^c^	>100 ^a^
**4**	>100 ^c^	>100 ^a^
**5**	>100 ^c^	>100 ^a^
**6**	>100 ^c^	>100 ^a^
**7**	>100 ^c^	>100 ^a^
**8**	66.35 ± 1.44 ^b^	>100 ^a^
**9**	43.08 ± 0.63 ^a^	>100 ^a^
**10**	40.56 ± 1.41 ^a^	>100 ^a^
**11**	>100 ^c^	>100 ^a^
**12**	>100 ^c^	>100 ^a^
**13**	>100 ^c^	>100 ^a^
**14**	>100 ^c^	>100 ^a^
**15**	>100 ^c^	>100 ^a^

Values represent means ± S.D*.* of three determinations. Values bearing different superscripts in the same column are significantly different (*p* < 0.05).

## 3. Experimental

### 3.1. General Methods

Sephadex LH-20 column (10–25 µm, GE Healthcare Bio-Science AB, Uppsala, Sweden) and Daisogel ODS-B with MPLC system (5 × 80 cm, Mitsubishi Chemical Co., Tokyo, Japan), 110UV/VIS detector (Gilson, Middleton, WI, USA) and TBP 5002 pump (Tauto Biotech, Shanghai, China) were used for column chromatography. TLC was carried out on pre-coated silica gel 60 F_254_ plates (Merck, Darmstadt, Germany); spots were detected under UV radiation (254 nm) and by spraying with FeCl_3_ solution and 10% H_2_SO_4_ followed by heating. The ^1^H- and ^13^C-NMR spectra were recorded at 300 MHz on a Gemini 2000 instrument (Varian, Palo Alto, CA, USA) and ^1^H-NMR, 600 MHz; ^13^C-NMR, 150 MHz on a Varian VNS (Varian, Palo Alto, CA, USA) and the resolution fast atom bombardment mass spectrum (LRFAB-MS) were measured with a JMSAX505WA instrument (JEOL, Tokyo, Japan).

### 3.2. Plant Material

The leaves of CL (1.8 kg) were collected from the Korea Forest Research Institute, Suwon, Korea in September 2010 and certified by Minwon Lee (Phamacognosy Lab, College of Pharmacy, Chung-Ang University). The voucher specimen (CL2010-9) was deposited at the herbarium of the College of Pharmacy, Chung-Ang University.

### 3.3. Extraction and Isolation

The dry leaves of CL (1.8 kg) were extracted for 72 h at room temperature with 80% aqueous MeOH (8.0 L). The concentrated extracts were subjected to preparative thin layer chromatography (TLC) using as solvent systems C:M:W (chloroform:MeOH:H_2_O = 6:4:1) or B:E:F (benzene:ethyl formate:formic acid = 1:7:1). The spots were detected under UV radiation (254 nm) and by spraying with FeCl_3_ and 10% H_2_SO_4_ or anisaldehyde-H_2_SO_4_ followed by heating. The CL aqueous layer (270 g) was concentrated and applied to a Sephadex LH-20 column (10–25 µm, 2 kg, 10 × 80 cm), and eluted with H_2_O containing increasing proportions of MeOH to afford from sub-fraction, 1 (134.6 g), 2 (23.5 g), 3 (10.9 g), 4 (33.3 g) and 5 (49.6 g). Repeated column chromatography of sub-fraction 2 on Daisogel ODS-B with MPLC system (300 μm, 500 g, 5 × 80 cm, Mitsubishi Chemical Co.), using a H_2_O:MeOH gradient yielded compounds **2** (481 mg), **4** (300 mg), **5** (15.5 g), **6** (87.5 mg). The sub-fraction 3 gave compounds **1** (370 mg), **3** (692 mg), sub-fraction 4 afforded compounds **7** (810 mg), **8** (4.2 g), **11** (1.1 g), **12** (10.3 g), **13** (126.3 mg), **14** (1.8 g) and **15** (475 mg). Finally, sub-fraction 5 contained compound **9** (458 mg) and **10** (11 g), respectively (Figure 1).

*3-Caffeoyl quinic acid Methyl ester* (**1**). Dark brown amorphous powder; Negative FAB-MS *m/z*: 367 [M−H]^−^, 179 [M−quinic acid methyl ester:caffeic acid]^−^. ^1^H-NMR (300 MHz, MeOH-*d_4_*): δ 2.17 (2H, m, H-6'), 2.22 (2H, m, H-2'), 3.75 (3H, s, OCH_3_), 3.77 (1H, m, H-4'), 4.19 (1H, m, H-5'), 5.33 (1H, m, H-3'), 6.29 (1H, d, *J* = 16.2 Hz, H-8), 6.90 (1H, d, *J* = 8.4 Hz, H-5), 7.06 (1H, dd, *J* = 0.6, 8.4 Hz, H-6), 7.18 (1H, d, *J* = 0.6 Hz, H-2), 7.57 (1H, d, *J* = 16.2 Hz, H-7).

*4-Caffeoyl quinic acid* (**2**). Dark brown amorphous powder; Negative FAB-MS *m/z*: 353 [M−H]^−^, 179 [M−quinic acid:Caffeic acid]^−^, ^1^H-NMR (300 MHz, MeOH-*d_4_*): δ 1.90 (2H, m, H-2'), 2.06 (2H, m, H-6'), 3.73 (1H, m, H-5'), 4.13 (1H, m, H-3'), 5.18 (1H, m, H-4'), 6.26 (1H, d, *J* = 16.8 Hz, H-8), 6.76 (1H, d, *J* = 7.8 Hz, H-5), 7.00 (1H, dd, *J* = 1.8, 7.8 Hz, H-6), 7.04 (1H, s, H-2), 7.48 (1H, d, *J* = 16.8 Hz, H-7).

*3-Caffeoyl quinic acid* (**3**). Dark brown amorphous powder; ^1^H-NMR (300 MHz, MeOH-*d_4_*): δ 2.20 (2H, m, H-6'), 2.24 (2H, m, H-2'), 3.77 (1H, m, H-4'), 4.22 (1H, m, H-5'), 5.37 (1H, m, H-3'), 6.29 (1H, d, *J* = 15.6 Hz, H-8), 6.87 (1H, d, *J* = 8.1 Hz, H-5), 7.13 (1H, dd, *J* = 1.8, 8.1 Hz, H-6), 7.15 (1H, d, *J* = 1.8 Hz, H-2), 7.57 (1H, *d*, *J* = 15.6 Hz, H-7).

*3-O-galloyl-β-d-glucopyranoside* (**4**). Yellow amorphous powder; Negative FAB-MS *m/z*: 331 [M−H]^−^, 169 [M−glucoside:Gallic aicd]^−^, ^1^H-NMR (300 MHz, MeOH-*d_4_*): δ 3.20 (1H, m, H-3'), 3.33 (1H, m, H-4'), 3.54 (2H, m, H-6', 5'), 3.76 (1H, m, H-6'), 3.81 (1H, m, H-2'), 5.57 (1H, d, *J* = 7.8Hz, H-1'), 7.07 (2H, s, H-2, 6), ^13^C-NMR (150 MHz, MeOH-*d_4_*): δ 60.8 (C-6'), 69.8 (C-4'), 73.2 (C-2'), 76.8 (C-3'), 78.5 (C-5'), 94.9 (C-1'), 109.3 (C-2. 6), 119.3 (C-1), 139.0 (C-4), 145.8 (C-3, 5), 165.0 (C-7).

*Bergenin* (**5**). White amorphous powder; Negative FAB-MS *m/z*: 327 [M−H]^−^, ^1^H-NMR (600 MHz, MeOH-*d_4_*): δ 3.47 (1H, t, 1H *J* = 9.3 Hz, H-3'), 3.73 (2H, m, H-11), 3.84 (1H, t, 1H, t, *J* = 8.1 Hz, H-2'), 3.89 (3H, s, OCH_3_), 4.04 (1H, t, *J* = 9.6 Hz, H-4'β), 4.09 (1H, t, *J* = 9.6 Hz, H-4'α), 4.96 (1H, d, *J* = 10.2 Hz, H-1'), 7.07 (1H, s, H-7), ^13^C-NMR (150 MHz, MeOH-*d_4_*): δ 59.4 (C-12), 61.2 (C-11), 70.4 (C-3'), 72.8 (C-10β), 74.2 (C-4'β), 80.0 (C-4'α), 81.7 (C-2'), 109.7 (C-7), 115.9 (C-10α), 118.0 (C-6α), 140.8 (C-9), 148.0 (C-10), 150.8 (C-8), 164.3 (C-6).

*Norbergenin* (**6**). White amorphous powder; ^1^H-NMR (300 MHz, MeOH-*d_4_*): δ 3.47 (1H, t, 1H *J* = 9.0 Hz, H-3'), 3.73 (2H, m, H-11), 3.84 (1H, t, *J* = 8.1 Hz, H-2'), 4.04 (1H, t, *J* = 9.6 Hz, H-4'β), 4.09 (1H, d, *J* = 9.6 Hz, H-4'α), 5.26 (1H, d, *J* = 10.2 Hz, anomeric H-1'), 7.08 (1H, s, H-7).

*11-Galloylbergenin* (**7**). White amorphous powder; Negative FAB-MS *m/z*: 479 [M−H]^−^. ^1^H-NMR (300 MHz, MeOH-*d_4_*): δ 3.70 (1H, t, *J* = 9.3, H-3'), 3.88 (3H, s, OCH_3_), 3.95 (2H, m, H-11), 4. 13 (1H, t, *J* = 10.2 Hz, H-2'), 4.30 (1H, dd, *J* = 3, 12 Hz, H-4'β), 4.66 (1H, dd, *J* = 12, 6,6 Hz, H-4'α), 5.05 (1H, d, *J* = 10.2 Hz, H-1'), 7.11 (1H, s, H-7), 7.18 (1H, s, H-2, 6), ^13^C-NMR (150 MHz, MeOH-*d_4_*): δ 60.3 (C-12), 63.7 (C-11), 70.2 (C-3'), 72.7 (C-10β), 73.8 (C-4'β), 79.1 (C-4'α), 80.0 (C-2'), 109.1 (C-7), 110.7 (C-7'), 116.1 (C-10α, 10'α), 118.6 (C-6α), 119.6 (C-6'α), 139.0 (C-9), 141.0 (C-9'), 145.6 (C-10, 10'), 148.2 (C-8), 151.0 (C-8'), 166.3 (C-6), 166.8 (C-6').

*Tellimagrandin I* (**8**). Dark yellow amorphous powder; Positive FAB-MS *m/z*: 787 [M+H]^+^, ^1^H-NMR (600 MHz, Aceton-*d_4_*+D_2_O): δ 3.79 (1H, dd, *J* = 13.2, 1.8 Hz, H-6"β), 3.87 (1H, dd, *J* = 13.2, 1.8 Hz, H-6"α), 4.28 (1H, dd, *J* = 6.6, 9.6 Hz, H-4"α), 4.69 (1H, dd, *J* = 6.6, 9.6 Hz, H-4"β), 5.11 (1H, m, H-5"α), 5.12 (1H, m, H-5"β), 5.14 (1H, d, *J* = 7.8 Hz, H-1"β), 5.25 (1H, dddd, *J* = 1.2, 10.2 Hz, H-2"α), 5.27 (1H, dddd, *J* = 1.2, 10.2 Hz, H-2"β), 5.29 (1H, t, *J* = 6.6 Hz, H-6"β), 5.31 (1H, t, *J* = 6.6 Hz, H-6"α), 5.57 (1H, d, *J* = 3.6 Hz, H-1"α), 5.62 (1H, t, *J* = 10.2, 9.6 Hz, H-3"β), 5.89 (1H, t, *J* = 10.2, 9.6 Hz, H-3"α), 6.49 (2H, s, HHDP-3'), 6.66 (2H, s, HHDP-3'), 6.95 (2H, s, G-2, 6), 6.99 (2H, s, G-2, 6), 7.05 (2H, s, G-2, 6), 7.06 (2H, s, G-2, 6), ^13^C-NMR (150 MHz, Aceton-*d_4_*+D_2_O): δ 63.0 (C-6"α, 6"β), 66.3 (C-5"α), 70.4 (C-4"α, 4"β), 70.6 (C-3"α, 2"α), 70.6 (C-5"β), 72.0 (C-3"β), 73.9 (C-2"β), 90.1 (C-1"α), 95.5 (C-1"β), 105.9 (HHDP-3), 106.0 (HHDP-3'), 109.1 (HHDP-1, 1'), 109.3 (G-1, 1'), 115.6 (G-2), 118.8 (G-2'), 124.3 (HHDP-5, 5'), 124.9 (HHDP-2, 2'), 135.6 (G-4'), 138.9 (G-4), 144.6 (HHDP-4, 4'), 144.8 (HHDP-6, 6'), 145.4 (G-3), 145.7 (G-3'), 165.5 (-COO ), 165.8 (-COO), 167.3 (-COO), 168.0 (-COO).

*Tellimagrandin II* (**9**). Dark yellow amorphous powder; Negative FAB-MS *m/z*: 953 [M−H]^−^, 169 [M−tellimagrandin I:Gallic acid]^−^, ^1^H-NMR (600 MHz, Aceton-*d_4_*+D_2_O): δ 3.92 (1H, d, *J* = 6.6 Hz, H-6"β) 4.58 (1H, dd, *J* = 1.2, 6.6 Hz, H-4"), 5.26 (1H, t, *J* = 8.4, 9.6 Hz, H-5"), 5.38 (1H, dd, *J* = 6.6 Hz, H-6"α), 5.64 (1H, dd, *J* = 1.2, 8.4 Hz, H-2"), 5.87 (1H, t, *J* = 8.4, 9.6 Hz, H-3"), 6.22 (1H, d, *J* = 8.4 Hz, H-1"), 6.68 (2H, s, HHDP-3'), 7.00 (2H, s, G-2, 6), 7.03 (2H, s, G-2, 6), 7.13 (2H, s, G-2, 6), ^13^C-NMR (150 MHz, Aceton-*d_4_*+D_2_O): δ 62.2 (C-6"), 69.8 (C-4"), 71.0 (C-2"), 72.2 (C-5"), 72.4 (C-3"), 92.8 (C-1"), 107.0 (HHDP-3), 107.2 (HHDP-3'), 109.2 (HHDP-1, 1'), 109.3 (G-1', 1",1"'), 114.8 (G-2"'), 118.8 (G-2', 2"), 124.8 (HHDP-2, 2'), 125.0 (HHDP-5, 5'), 135.6 (G-4", 4"'), 138.9 (G-4'), 144.6 (HHDP-4, 4'), 144.9 (HHDP-6, 6'), 145.1 (G-3", 3"'), 145.3 (G-3'), 164.2 (-COO), 165.0 (-COO), 165.6 (-COO), 166.9 (-COO), 167.3 (-COO).

*Casuarinin* (**10**). Dark yellow amorphous powder; Positive FAB-MS *m/z*: 937 [M+H]^+^, ^1^H-NMR (600 MHz, Aceton-*d_4_*+D_2_O): δ 3.72 (1H, t, *J* = 13.2 Hz, H-6"β), 3.79 (1H, t, *J* = 13.2 Hz, H-6"α), 4.43 (1H, dd, *J* = 6.6, 9.6 Hz, H-4"α), 4.46 (1H, dd, *J* = 6.6, 9.6 Hz, H-4"β), 4.83 (1H, m, H-5"α), 4.88 (1H, m, H-5"β), 4.79 (1H, m, H-2"α) 4.82 (1H, m, H-2"β), 4.83 (1H, d, *J* = 7.8 Hz, H-1"β), 4.96 (1H, m, H-6"β), 4.98 (1H, m, H-6"α), 5.32 (1H, d, *J* = 3.6 Hz, H-1"α), 5.47 (1H, t, *J* = 10.2, 9.6 Hz, H-3"β), 5.66 (1H, t, *J* = 10.2, 9.6 Hz, H-3"α), 5.90 (2H, s, G-2, 6), 6.26 (2H, s, HHDP-3), 6.81 (2H, s, HHDP-3), 6.86 (2H, s, HHDP-3), 6.92 (2H, s, HHDP-3), ^13^C-NMR (150 MHz, Aceton-*d_4_*+D_2_O): δ 63.0 (C-6"α, 6"β), 66.2 (C-5"α), 70.3 (C-4"α, 4"β), 70.4 (C-3"α, 2"α), 70.8 (C-5"β), 72.0 (C-3"β), 72.5 (C-2"β), 90.2 (C-1"α), 95.4 (C-1"β), 103.2 (HHDP-3), 105.6 (HHDP-3'), 108.6 (HHDP-1), 109.1 (HHDP-1'), 109.3 (G-1, 1'), 115.5 (G-2), 118.8 (G-2'), 124.0 (HHDP-5, 5'), 124.2 (HHDP-2, 2'), 135.5 (G-4'), 139.0 (G-4), 142.8 (HHDP-4, 4'), 144.7 (HHDP-6, 6'), 145.5 (G-3), 145.7 (G-3'), 165.6 (-COO), 165.8 (-COO), 167.3 (-COO), 168.0 (-COO).

*Quercetin* (**11**). Yellow amorphous powder; ^1^H-NMR (300 MHz, DMSO-*d_6_*+D_2_O): δ 6.19 (1H, d, *J* = 2.1 Hz, H-6), 6.41 (1H, d, *J* = 1.8 Hz, H-8), 6.89 (1H, d, *J* = 8.4 Hz, H-5'), 7.55 (1H, dd, *J* = 2.1, 8.4 Hz, H-6'), 7.68 (1H, d, *J* = 2.1 Hz, H-2').

*Quercitrin* (**12**). Yellow amorphous powder; Negative FAB-MS *m/z*: 447 [M−H]^−^, 301 [M−rhamnose:Quercetin]^−^, ^1^H-NMR (300 MHz, DMSO-*d_6_*+D_2_O): δ 0.80 (3H, d, *J* = 6.6 Hz, H-6"), 3.18 (3H, m, H-3", 5", 4"), 3.97 (1H, dd, *J* = 4.5 Hz, H-2"), 5.24 (1H, s, H-1"), 6.22 (1H ,d, *J* = 1.8 Hz, H-6), 6.41 (1H, d, *J* = 1.8 Hz, H-8), 6.88 (1H, d, *J* = 8.4 Hz, H-5'), 7.26 (1H, dd, *J* = 2.1, 8.4 Hz, H-6'), 7.29 (1H, d, *J* = 2.1 Hz, H-2').

*Quercetin 3-O-β-d-glucuronide* (**13**). Yellow amorphous powder; Negative FAB-MS *m/z*: 477 [M−H]^−^, 301 [M−glucuronide:Quercetin]^−^, ^1^H-NMR (300 MHz, DMSO-*d_6_*+D_2_O): δ 3.08 (2H, m, H-6"), 3.21 (1H, m, H-2"), 3.33 (2H, m ,H-4", 3"), 3.58 (1H, m, H-5"), 5.48 (1H, d, *J* = 7.8 Hz, H-1"), 6.22 (1H, d, *J* = 2.1 Hz, H-6), 6.42 (1H, d, *J* = 2.1 Hz, H-8), 6.87 (1H, d, *J* = 8.7 Hz, H-5'), 7.57 (1H, dd, *J* = 2.1, 8.7 Hz, H-6'), 7.59 (1H, d, *J* = 2.1 Hz, H-2').

*Datiscetin 3-O-β-d-rhamnpyranoside* (**14**). Yellow amorphous powder; Positive FAB-MS *m/z*: 447 [M−H]^−^, 283 [M−rhamnose:Datiscetin]^−^, ^1^H-NMR (300 MHz, DMSO-*d_6_*+D_2_O): δ 0.83 (3H, d, *J* = 6.6 Hz, H-6"), 3.18 (3H, m, H-3", 5", 4"), 3.96 (1H, dd, *J* = 4.5 Hz, H-2"), 5.24 (1H, s, H-1"), 6.22 (1H, d, *J* = 1.2 Hz, H-6), 6.42 (1H, d, *J* = 1.2 Hz, H-8), 6.88 (2H, d, *J* = 7.6, 8.4 Hz, H-3', 5'), 7.29 (1H, dd, *J* = 7.6, 8.4 Hz, H-4'), 7.59 (1H, d, *J* = 7.5 Hz, H-6').

*Myricetin 3-O-β-d-rhamnopyranoside* (**15**). Yellow amorphous powder; Negative FAB-MS *m/z*: 447 [M−H]^−^, 317 [M−rhamnose:Myricetin]^−^, ^1^H-NMR (300 MHz, DMSO-*d_6_*+D_2_O): δ 0.81 (3H, d, *J* = 6.6 Hz, H-6"), 3.15 (1H, d, *J* = 9.4, H-5"), 3.16 (1H, t, *J* = 9.4, 6.1, H-, 4"), 3.32 (1H, dd, *J* = 9.4, 3.3 Hz, H-3"), 3.97 (1H, bs, *J* = 1.4 Hz, H-2"), 5.19 (1H, s, H-1"), 6.39 (2H, d, *J* = 1.2 Hz, H-6, 8), 6.88 (2H, s, H-2', 6').

### 3.4. Antioxidant Activity

#### 3.4.1. Measurement of DPPH Radical Scavenging Activity

Each sample in absolute EtOH was added to DPPH solution (0.1 mM, in absolute EtOH). After mixing gently for 30 min, optical densities were measured at 518 nm using a microplate reader (TECAN, Salzburg, Austria). L-Ascorbic acid (Sigma, St. Louis, MO, USA) was used as a positive control [21].

#### 3.4.2. Xanthine Oxidase Superoxide Scavenging Activity

After each sample was added to 50 mM phosphate buffer (pH 7.5) containing 0.05 mM EDTA, 0.2 mM hypoxanthine and 0.1 mM NBT, xanthine oxidase (1.2 U/μL) was then added to this mixture. After mixing gently for 10 min, optical densities were measured at 612 nm using a microplate reader (Tecan). Allopurinol was used as a positive control [22].

### 3.5. Anti-Proliferation and Cytotoxicity Assays

#### 3.5.1. Cell Culture

RAW 264.7 macrophage, DU-145 and LNCaP human prostate cancer cell lines were purchased from the Korean Cell Line Bank. The RAW 264.7 macrophage cell and human prostate cancer cell lines was grown at 37 °C in a humidified atmosphere (5% CO_2_) in DMEM (Sigma) and RPMI (Sigma) containing 10% fetal bovine serum, 10 IU/mL penicillin G and 100 μg/mL streptomycin (Gibco BRL, Grand Island, NY, USA).

#### 3.5.2. Measurement of Cell Viability

After culturing of RAW 264.7 macrophage (3.5 × 10^5^ cells/200 μL medium) in 96-well plates and incubating for 2 h, in 96-well plates and incubating for 24 h, the cells were treated with the test samples. The cells were incubated for an additional 24 h, and the medium was replaced with fresh medium containing 0.5 mg/mL 3-(4,5-dimethylthiazol-2-yl)-2,5-diphenyltetrazolium bromide (MTT) (Sigma). Incubation was continued for 4 h at 37 °C. The medium was then removed and the MTT-formazan produced was dissolved in dimethyl sulfoxide (DMSO). The extent of the reduction of MTT to dark purple crystals within the cells was quantified by measuring the absorbance at 540 nm using the microplate reader (Tecan) [24].

#### 3.5.3. Anti-Proliferation Assays

After culturing of DU145 and LNCaP human prostate cancer cell lines (1 × 10^4^ cells/ 200 μL medium) in 24-well plates and incubating for 24 h, the cells were treated with the test samples. Plates were incubated for 72 h after which bioassays were performed. LNCaP cells were handled in a similar manner, and the medium was replaced with fresh medium containing 0.5 mg/mL 3-(4,5-dimethylthiazol-2-yl)-2,5-diphenyltetrazolium bromide (MTT) (Sigma). Incubation was continued for 4 h at 37 °C. The medium was then removed and the MTT-formazan produced was dissolved in dimethyl sulfoxide (DMSO). The extent of the reduction of MTT to dark purple crystals within the cells was quantified by measuring the absorbance at 540 nm using the microplate reader (Tecan) [24].

### 3.6. Statistical Analysis

All data are expressed as mean ± S.D. Values were performed by one-way analysis of variance (ANOVA) followed by Student-Newman-Keuls (S-N-K) test using the SPSS software package; the values were considered significantly different when the *p* value was less than 0.05.

## 4. Conclusions

Fifteen phenolic compounds **1**–**15**, inclusing three caffeoyl derivatives **1**–**3**, four gallotannins **4**–**7**, three ellagitannins **8**–**10** and five flavonoids **11**–**15** were isolated from CL. DPPH radical scavenging and superoxide scavenging activity, as well as, anti-proliferative activity on prostate cancer call lines (DU145 and LNCaP) of the isolated compounds were evaluated. As a results, the ellagitannin derivatives **8**–**10** showed potent anti-oxidative and anti-proliferative activities on prostate cancer cell lines. These results suggested that CL extract and its phenolic compounds could potentially be developed as ingredients with anti-oxidative and androgen sensitive anti-proliferation activity.
